# Analysis and Thoughts about the Negative Results of International Clinical Trials on Acupuncture

**DOI:** 10.1155/2015/671242

**Published:** 2015-06-16

**Authors:** Wei-hong Liu, Yang Hao, Yan-jing Han, Xiao-hong Wang, Chen Li, Wan-ning Liu

**Affiliations:** Institute of Acupuncture and Moxibustion, China Academy of Chinese Medical Sciences, Beijing 100700, China

## Abstract

An increasing number of randomized controlled trials (RCTs) of acupuncture have proved the clinical benefits of acupuncture; however, there are some results that have shown negative results or placebo effects. The paper carried out an in-depth analysis on 33 RCTs in the 2011 SCI database, the quality of the reports was judged according to Jadad scores, and the “Necessary Information Included in Reporting Interventions in Clinical Trials of Acupuncture (STRICTA 2010)” was taken as the standard to analyze the rationality of the therapeutic principle. The difference between the methodology (Jadad) scores of the two types of research reports did not constitute statistical significance (*P* > 0.05). The studies with negative results or placebo effects showed the following deficiencies with respect to intervention details: (1) incompletely rational acupoint selection; (2) inconsistent ability of acupuncturists; (3) negligible needling response to needling; (4) acupuncture treatment frequency too low in most studies; and (5) irrational setting of placebo control. Thus, the primary basis for the negative results or placebo effects of international clinical trials on acupuncture is not in the quality of the methodology, but in noncompliance with the essential requirements proposed by acupuncture theory in terms of clinical manipulation details.

## 1. Introduction

As an integral part to the Chinese medical and health care system, acupuncture therapy is widely applied in clinical applications, effective in treatment, economical, and safe and thereby generally accepted by Chinese people. Since the sixth century AD, acupuncture has successively spread to various countries of the world, making considerable contributions to relieving people from diseases worldwide. Along with the development of evidence-based medicine, international clinical trials on acupuncture have been increasing in number and raising greater controversies on whether or not acupuncture is effective. While the majority of international clinical trial reports on acupuncture have demonstrated that acupuncture therapy is indeed effective, some research has shown that acupuncture therapy benefits patients, but is equivalent to the placebo effect [1], and some people consider acupuncture therapy to be ineffective [2]. Currently, it is widely believed that such a result is a product of the higher-quality methodology for international randomized controlled trials (RCTs) of acupuncture. The purpose of the current study was to determine the basis for the negative results or placebo effects in published acupuncture RCTs from the perspective of methodology and interventions after comprehensively reading and analyzing the published acupuncture RCTs retrieved from the 2011 SCIE database, with the exception of research conducted in China.

## 2. Materials and Methods

### 2.1. Search Strategy

The computer retrieval was carried out in “Science Citation Index Expanded (SCIE)” and the retrieval type was “(‘acupuncture' [MeSH Terms] OR ‘acupuncture' [All Fields] OR ‘acupuncture therapy' [MeSH Terms] OR (‘acupuncture' [All Fields] AND ‘therapy' [All Fields]) OR ‘acupuncture therapy' [All Fields] OR (‘moxibustion' [MeSH Terms] OR ‘moxibustion' [All Fields] AND ‘2011/1/1' [PDat]: ‘2011/12/31' [PDat] AND English [lang]).”

### 2.2. Inclusion Criteria

The inclusion criteria were as follows: (1) randomized controlled acupuncture, acupressure, or moxibustion trials published in 2011 from the SCIE database; (2) patients underwent the trial regardless of age, gender, ethnicity, or course of disease; and (3) intervention of the observation or controlled group was based on the theory of meridians and collaterals and the patients were treated by acupuncture, acupressure, and/or moxibustion.

### 2.3. Exclusion Criteria

The exclusion criteria were as follows: (1) nonrandomized trials; (2) nonclinical trials; (3) the intervention did not conform to the objective of the research; (4) duplicated articles; and (5) the first author was from China or the trial was conducted in China.

### 2.4. Data Collection and Analysis

Evaluation was performed independently by two authors (Yang Hao and Wan-ning Liu). Relevant full articles were sorted and cross-examined. Any discrepancies were discussed or further evaluated by a 3rd author (Wei-hong Liu). Methodology was evaluated based on the Jadad score [3]. The specific evaluation standard is shown in Table 1.

## 3. Results

### 3.1. Articles Included

**Figure 1:**
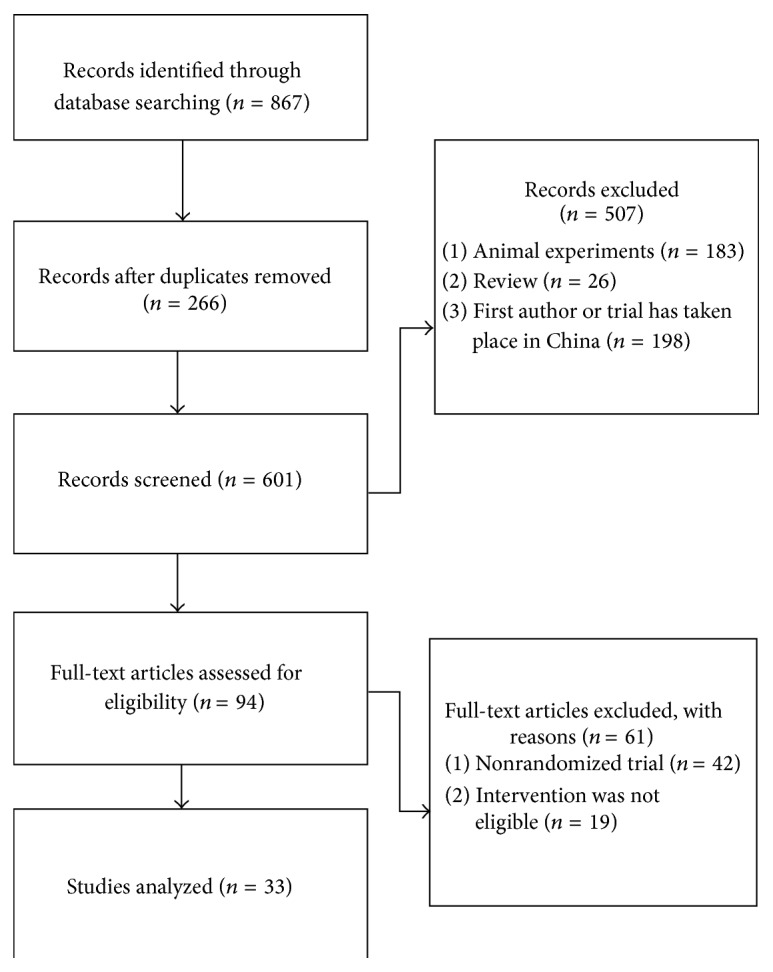
The flow diagram of the research.

Of the 867 articles retrieved, 33 studies [2, 4–35] met the inclusion criteria for the current analysis (Figure 1).

### 3.2. Features of the Studies

Features of the included trials are detailed in Table 2.

### 3.3. Jadad Score of the Trials

According to the research results, the 33 reports were classified into two types (positive results and negative results or placebo effects). The 33 reports were read and the key points were extracted. According to the Jadad score, the lowest methodology quality was scored 0, while the highest methodology quality was scored 5. The clinical trial was considered low in quality if the score was ≤2 and was considered high in quality if the score was ≥3. The Jadad scores of the research report methodologies are shown in Table 3, and the Jadad score comparison of the acupuncture RCT methodologies is shown in Table 4.

By adoption of SPSS 13.0, data in Table 4 was subjected to a *χ* ^2^ test; the difference between the two groups was not statistically significant (*P* = 1.0). The quality of the clinical trial report methodology on acupuncture with positive results is similar to the clinical trial reports with negative results or placebo effects, which indicates that the difference in quality of the methodology is not the primary reason for the different clinical research results of acupuncture.

### 3.4. Analysis of Acupuncture RCTs Intervention Details with Positive Results, Negative Results, and Placebo Effects

The intervention details of the RCTs with positive results, negative results, and placebo effects were compared. Due to the small number of RCTs with negative results or placebo effects collected in 2011, the acupuncture RCTs from 2005 to 2010 in the SCIE database were retrieved. The retrieval terms were as follows: “acupuncture” [MeSH terms] OR “acupuncture” [all fields] OR “acupuncture therapy” [MeSH terms] OR “acupuncture” [all fields] AND “therapy” [all fields] OR “acupuncture therapy” [all fields] OR “moxibustion” [MeSH terms] OR “moxibustion” [all fields] AND “2005/1/1” [PDat]: “2010/12/31” [PDat] AND randomized controlled trial [PDat] AND English [lang]. Seven reports [36–42] with negative results or placebo effects were analyzed together with the 10 reports with negative results or placebo effects.

According to the “Necessary Information Included in Reporting Interventions in Clinical Trials of Acupuncture (STRICTA 2010),” the authors designed an intervention table for the RCTs and compared the intervention details of the RCTs with positive results, negative results, and placebo effects. The detailed information of the reports is shown in Tables 5 and 6.

It is known that the clinical treatment process of acupuncture involves not only the operation of acupuncture–moxibustion therapy, but also the rational selection of therapeutic principles and methods, rational application of acupoints, manipulation, and the correct setting of the therapy, and so on. It is apparent from the analysis of items displayed in Tables 5 and 6 that the intervention process in the negative results and placebo effects was replete with defects.

#### 3.4.1. Interventions of Some Trials Are Improper

To select a proper therapeutic method is the key to assuring a curative effect; however, the authors showed that some interventions in the 17 reports were improper. For example, in item 001, moxibustion was adopted to treat constipation; in item 009, which involved the treatment of postmenopausal women suffering knee joint pain, women without medical knowledge performed acupressure by themselves at home; and in item 005, when treating the nausea and vomiting associated with labor and delivery, only a wrist band that slightly stimulates PC 6 was used. Despite certain therapeutic function, these measures are all not the most proper choice. For example, constipation is most often treated clinically with acupuncture; however, for excess syndrome or heat syndrome constipation, it is improper to use moxibustion. Similarly, it is doubtful that the wrist band which stimulates PC 6 is satisfactory to achieve a therapeutic effect like acupuncture. With respect to the studies with positive results, the applied methods were effective intervention, such as filiform needles, electroacupuncture, blunt needles, or auricular acupuncture. Clearly, effective intervention is an important factor for the results of the trial.

#### 3.4.2. Acupoint Selection in Some Studies Is Not Completely Rational

Each acupoint has its therapeutic effect. Rich experiences in acupuncture have been accumulated through the inheritance for thousands of years in China. For example, the most effective acupoints for constipation treatment are ST25, ST36, ST28, ST29, and TE6, while the researcher for item 001 selected ST23 and ST27. Clinically, ST23 is often involved in the treatment of gastric diseases or mental diseases, such as vexation and manic-depressive psychosis, while ST27 is mainly used in the treatment of hypogastrium distention and fullness, difficult urination, hernia, spermatorrhoea, premature ejaculation, and other male diseases. The selection of improper acupoints results in a low clinical curative effect. In item 003, the researcher only selected LI4 to treat infantile colic. LI4 belongs to the large intestine meridian and plays a role in treating intestinal disease, but it is clinically known for its effect on head/face diseases, sweating disorders, and gynecologic diseases, including menstrual disorders, vaginal discharge, and parturition. If GV12 and ST36 are added in the prescription, the effect would be significantly improved. Thus, the researcher may select a single acupoint by reducing confounding factors in interventions as much as possible, but not considering that acupuncture therapy needs the compatibility of acupoints for enhancement of effect and improvement of curative effect in a practical environment. The acupoints selected in the studies with positive results were correct and most effective according to clinical experiences. The comparison indicated that the rational selection of acupoints is directly related to the validity of the trial.

#### 3.4.3. Needling Response Is Neglected in Most Studies

The famous acupuncture work,* Biaoyoufu,* in ancient China says, “Quick needling response results in quick action, otherwise the late needling response causes treatment failure,” which means that the patients' meridian-*qi* circulation should be considered when needling to realize the needling response. Of 17 reports with negative results or placebo effects, 65% (11/17) did not mention whether or not the needling response was achieved. This is a problem that should be addressed by the research design and execution staff. The physicians of acupuncture and moxibustion in China mostly have such experiences that immediately after needle insertion they must observe the patient's response, earnestly feel the sense beneath the needle tip, and repeatedly operate the needle body so that the endurable feelings of sourness, numbness, swelling, heaviness, and pain can be felt by the patients. Meanwhile, the operator also feels heaviness and tightening beneath the needle tip, which is called the needling response. If such a feeling is generated, a good curative effect can be realized, whereas if it is not, the effect is slow or not apparent. As an intervention in the observation group, no needling response suggests no real or ineffective stimulation to the acupoint. In this way, the curative effect will be reduced greatly and a negative result may be more likely to occur. It is noteworthy that 65% (15/23) of the studies with positive results did not mention whether or not the needling response was achieved. As a complicated intervention, the effectiveness of acupuncture is influenced by multiple factors.

#### 3.4.4. The Requirements of the Acupuncturist Is Neglected in Many Studies

As shown in Table 5, acupuncturists involved in the clinical trials had inconsistent qualifications. The proportion of excellent acupuncturists in the studies with negative results or placebo effects was 65% (11/17). Some of the acupuncturists work part-time and are actually nurse practitioners (003), some have achieved the lowest requirements (008), some are midwives without acupuncturist qualifications (006), and some ask the patients to operate on themselves at home (004, 009, and 012). Because of low-level acupuncturists and such simple treatments, it is really difficult to fully realize the curative effect of acupuncture. The proportion of excellent acupuncturists in the studies with positive results was 74% (17/23), which was significantly higher than the studies with negative results and placebo effects. Acupuncture is a therapeutic method that has high technical skill requirements. He [43] concluded that the proficiency and level of clinical acupuncture skill constitute decisive factors of a clinical curative effect, as well as the advantages of famous veteran physicians of traditional Chinese medicine. Inexperienced or unqualified acupuncturists undoubtedly lower the effectiveness and safety of acupuncture treatment, especially when patients are asked to treat themselves.

#### 3.4.5. The Acupuncture Treatment Frequency Is Too Low in Most Studies

Among the 17 studies listed in Table 5, eight had a treatment frequency of 1-2 times/week (002, 003, 005, 007, 008, 011, 013, and 016), accounting for 47% of the studies; 53% of the studies had a treatment frequency ≥3 times/week (001, 014, and 017). Among the studies with positive results, eight had a treatment frequency of 1-2 times/week, accounting for 35%; 65% of studies with positive results had a treatment frequency ≥3 times/week. Indeed, the studies with positive results had a significantly higher treatment frequency. According to Cai and Ma [44], the influence of acupuncture at BL23 on urinary function peaks after the acupuncture is implemented for 1 hour and then slowly declines and recovers to the original level, with the effect lasting 2–6 hours. This finding is consistent with the metabolic principles in the human body. The curative effect of acupuncture is determined by the duration of the acupuncture effect remaining in the human body and the accumulation of multiple therapeutic effects. Therefore, the best treatment frequency of acupuncture is 1-2 times per day. In the event of one treatment per 2 days or an extended interval of time, it takes more time to accumulate the acupuncture effect, leading to a slower onset of effect. Moreover, different diseases require different treatment frequencies; for chronic diseases and permanent symptoms, the treatment frequency should be higher, and for chronic neuralgia (001), irritable bowel syndrome (011), and smoking cessation (017), it is evident that a good effect is difficult to realize if the frequency is one time per week.

In addition to all the factors above, based on the research demonstrating a clinical curative effect, the diseases which are best to be treated are selected. For example, for smoking cessation, a worldwide problem which is difficult to eradicate, if acupuncture is adopted at a frequency of one time per week, the effect is weak.

### 3.5. Reflections on Placebo Acupuncture Settings

The 17 studies with negative results or placebo effects are generated in comparison with other therapeutic measures. At the same time, the suitability of the control settings is also worthy of further analysis. The authors have analyzed the control setting list (Table 7) in these research reports and divided the control methods into the following three types: (1) no penetration into the skin (the Park sham needle) or heat insulation acupuncture; (2) slight penetration into the skin or press; and (3) stimulation of the nonacupoint parts. These three points will be analyzed one-by-one as follows.

#### 3.5.1. No Penetration into the Skin as a Control

Park sham acupuncture instruments were used in items 006, 013, 014, and 015, which is the control that did not penetrate into the skin. The instrument incorporates a round and blunt needle head which can be retracted into the needle handle and does not penetrate into the skin when the needle is touching the skin. The outer surface of the needle is fixed by double-faced adhesive tape and equipped with a small pipe to prevent the patient from seeing the truth. Park et al. [45] reported that the needle head would inevitably stimulate the skin and have a vivid effect on the skin, which will result in a physiologic effect. The Park sham acupuncture changes the method and tools of stimulation; thus the control method can also generate some therapeutic effect, but the researcher considers it as the control measure that cannot generate an effect or only shows a placebo effect. Therefore, when the measures of the observation group indicate the same curative effect as that of the control group, the measure of the observation group is considered to be invalid or have placebo effect only. The measure of the control group has some therapeutic effect, so the result of the observation group is false-negative. The Park sham acupuncture method is similar to a pressing method. The acupressure is referred to as the “indicator” in acupuncture theory and exclusively used for infants, people afraid of acupuncture, nervous patients, or when the needle is lacking; acupressure is also a simple method with a treatment effect.

#### 3.5.2. Slight Penetration into the Skin as a Control

The 016 and 017 studies carried out the control using the shallow stimulation method; however, in clinical acupuncture and moxibustion, shallow acupuncture itself is an effective therapeutic method. The* Miraculous Pivot* has recorded that the light stimulation just stimulates the skin, while the semipenetration involves the skin, but not the muscle. The* A-B Classic of Acu-moxibustion* has clearly described that 14 acupoints can be penetrated by one* fen* (approximately 3.3 mm) and 20 acupoints can be penetrated by 2* fen* (approximately 6.6 mm) [46]. Another study [47] indicates that 42 patients with wrist myofascial pain were randomly distributed to the deep acupuncture group and the shallow acupuncture group with the same acupoints, and the acupuncture depth for the deep acupuncture group was 1.5 cm compared to 2 mm for the shallow acupuncture group. The McGill pain questionnaire was used as the evaluating indicator. The scores for the two groups before the treatment were 35.4 ± 14.53 and 34.75 ± 11.43, respectively, compared to 14.54 ± 10.88 and 22.25 ± 16.08 after the treatment. The results indicate that the two groups can relieve the pain, and the curative effect was not statistically different. Therefore, with respect to pain-related disease and disease suitable for shallow acupuncture, shallow acupuncture is unsuitable for the acupuncture control without a curative effect because it will result in false-negative properties of the observation group.

#### 3.5.3. Nonacupoint and Nonmeridian Acupoint as Controls

The researchers performing seven studies (002, 011, 012, 014, 015, 016, and 017) avoid or depart the known acupoints and meridians as the placebo control. The question involves how many acupoints the people have in their body. The WHO has approved that there are 361 meridian points and 48 extraordinary points; however, >2200 extraordinary acupoints have been collected [48] with formal names and main functions. Owing to all types of unfixed* a* shì points, it is easy to avoid the meridians, but hard to avoid the acupoints when designing the nonacupoint and nonmeridian controls. The parts avoiding the familiar meridians and acupoints are just defined as the nonmeridian and nonacupoint parts [49]. Furthermore, the area of the acupoints has not been measured until now, and the distance between the meridian or acupoint and the nonmeridian and nonacupoint part has not been determined. Therefore, the control with nonmeridian and nonacupoint parts is highly possible to apply the “point” with a therapeutic effect as the control, and the result of the observation group has a high possibility of a false-negative.

In summary, all of the three above-mentioned control methods showed a therapeutic effect; however, the researchers only think the therapeutic effect was from the placebo control and when the therapeutic effect of the observation group is similar to that of the control group, the conclusion is incorrect that the observation group therapy had no effect or was equal to the placebo. The other reason for the researcher to design the placebo control like this is possibly related to the “blind.” In view of the particularity of acupuncture, it is impossible to identify the placebo therapy meeting the blind requirement and being similar to acupuncture. Many experts [50–52] have written articles to discuss the methods of setting the control group in acupuncture RCTs; however, Liu [53] suggests using modern medical methods as the standard control and aiming at the most effective and most advanced method in mainstream medicine to directly discover the advantage or disadvantage of acupuncture and give full attention to the medical development of acupuncture.

## 4. Discussion

By analyzing acupuncture RCTs in the SCI database, it is discovered that the methodologic quality of research with positive results is not different from that of research with negative results or placebo effects. The methodologic quality is not the primary reason contributing to the difference in research results; however, each study with negative results or placebo effects has disadvantages on the intervention side, such as incomplete rational acupoint selection, inconsistent ability of acupuncturists, negligence of the needling response to needling, low frequency of the acupuncture treatment, and irrational setting of placebo control. Those directly weaken the positive property of the results in the observation group, and the setting of the placebo acupuncture control is opposite to the theory of acupuncture. The placebo acupuncture method has certain therapeutic effects instead of purely a placebo effect, thereby causing the false-negative property of the results in the observation group. It was shown that the sham acupuncture (placebo acupuncture) in the current acupuncture RCTs and the placebo control method was not reached by consensus. The Society of Acupuncture and Moxibustion gradually found that the clinical trials under ideal conditions are not suitable for acupuncture and moxibustion. Seeking the clinical research methods in the practical world, practical clinical research may be able to break the limit of the placebo acupuncture control and find the advantage of acupuncture therapy.

We can see that the current clinical research for acupuncture and moxibustion still reflects many methodologic problems and is not mature in terms of theory and practices. It is necessary to establish a clinical research method for acupuncture and moxibustion to meet the requirements of the acupoint theory, practice features, and clinical trials so that the clinical trial results for acupuncture and moxibustion are scientific, comply with medical ethics, completely meet the treatment effect advantages of acupuncture, and promote acupuncture to mainstream medicine.

The limitations of the research are as follows: (1) the research report is of limited duration, thus this paper inevitably suggests selection bias; (2) a common phenomenon exists in the sector that the probability of publishing of a negative article is lower than for a positive article, which will cause bias to the research conclusion; and (3) the Jadad scale is used to evaluate the methodologic quality of the article. The greatest strength of the scale is directly evaluating the verified test features related to the bias in the test effect evaluation, which is simple and clear; however, the Jadad score will be too general and arbitrary if most of the research is not defined, whether or not they are random or double blind.

## Figures and Tables

**Table 1:** Jadad score standard.

Items	Score standard		
	0	1	2
Randomization	Not randomized or inappropriate method of randomization	The study was described as randomized	The method of randomization was described and it was appropriate
Double blinding	Not blind or inappropriate method of blinding	The study was described as double blind	The method of double blinding was described and it was appropriate
Withdraws and drop outs	Not describing the follow-up	A description of withdraws and dropouts	—

**Table 2:** Features of the studies.

Number	Title	Disease	Intervention of the observation group	Intervention of the control group	Sample size	Outcome	Follow-up phase
001	“The Effectiveness of Moxibustion for the Treatment of Functional Constipation: A Randomized, Sham-Controlled, Patient Blinded, Pilot Clinical Trial”	Constipation	Moxibustion	Heat insulation moxa-moxibustion (place a heat insulator under the true moxa-moxibustion)	26	Invalid	2 weeks
002	“The efficacy of Acupressure at the Sanyinjiao Point in the Improvement of Women's General Health”	Woman health	Acupressure at SP 6	Sham acupuncture	86	Both valid, the real is the better	No mention
003	“Preliminary Research Article Electroacupuncture Is Not Effective in Chronic Painful Neuropathies”	Chronic neuralgia	Electroacupuncture for 12 weeks	Press the nonacupoint (not touch the tendo calcaneus in any meridian vessel on the back of leg)	16	Invalid	No mention
004	“Feeding, Stooling and Sleeping Pattern in Infants with Colic- A Randomized Controlled Trial of Minimal Acupuncture”	Infantile colic	Acupuncture	Blank	90	The symptoms improved, but no statistical difference	No mention
005	“The Effect of Acupuncture on Psychosocial Outcomes for Women Experiencing Infertility: A Pilot Randomized Controlled Trial”	Women experiencing infertility	Acupuncture	No intervention	32	Valid	No mention
006	“Effect of Acupuncture on Nausea and/or Vomiting during and after Cesarean Section in Comparison with Ondansetron”	Nausea and vomiting when delivery	Wrist band stimulative to acupoint	Wrist band nonstimulative to PC 6	450	Invalid	No mention
007	“Acupuncture in Children and Adolescents with Bronchial Asthma: A Randomised Controlled Study”	Bronchial asthma	Acupuncture	Conventional treatment of western medicine	93	Partial response	4 months
008	“True and Sham Acupuncture Produced Similar Frequency of Ovulation and Improved LH to FSH Ratios in Women with Polycystic Ovary Syndrome”	Polycystic ovarian syndrome	Electroacupuncture	Stick on the skin by sham needle tubing and adjust the current to 0	96	Both valid	3 months
009	“Effects of Auricular Acupuncture on Anthropometric, Lipid Profile, Inflammatory and Immunologic Markers: A Randomized Controlled Trial Study”	Obesity	Auricular acupuncture	Sham auricular acupuncture	204	Valid	No mention
010	“Acupuncture for “Frequent Attenders” with Medically Unexplained Symptoms: A Randomised Controlled Trial (CACTUS Study)”	Neurosis	Acupuncture	Conventional treatment	80	Valid	52 weeks
011	“The Effects of Acupuncture on the Inner Ear Originated Tinnitus”	Inner ear originated tinnitus	Acupuncture	Sham acupuncture (the Park sham acupuncture instrument)	63	Someone has valid in a short time	No mention
012	“Evaluation of Wet-Cupping Therapy for Persistent Non-Specific Low Back Pain: A Randomised, Waiting-List Controlled, Open-Label, Parallel-Group Pilot Trial”	Persistent nonspecific low back pain	Wet-cupping + excise + analgesic-antipyretic	Excise + analgesic-antipyretic	32	Valid	2 weeks
013	“Acupuncture in Preterm Babies during Minor Painful Procedures”	Acupuncture	Acupuncture	Without any measure	10	Valid	No mention
014	“Moxibustion for Cephalic Version: A Feasibility Randomised Controlled Trial”	Malposition	Moxibustion	Without any measure	20	Invalid	No mention
015	“Patient Education Integrated with Acupuncture for Relief of Cancer-Related Fatigue Randomized Controlled Feasibility Study”	Cancer-related fatigue	Patient education integrated with acupuncture	Self-management	13	Valid	No mention
016	“Effectiveness of Acupressure and Acustimulation in Minimizing Driving Simulation Adaptation Syndrome”	Minimizing driving simulation adaptation syndrome	Acupressure and acustimulation	Sham wristband stimulation	25	Valid	No mention
017	“Comparative Effects of Acupressure at Local and Distal Acupuncture Points on Pain Conditions and Autonomic Function in Females with Chronic Neck Pain”	Chronic neck pain	Acupuncture at local points	Acupuncture at distal points and blank control	33	Valid and the local points intervention is better	No
018	“Electroacupuncture for Cervical Ripening Prior to Labor Induction: A Randomized Clinical Trial”	Cervical ripening prior to labor induction	Electroacupuncture	Moxibustion	72	Valid	No mention
019	“Effects of Acupuncture in Reducing Attrition and Mortality in HIV-Infected Men with Peripheral Neuropathy”	HIV-infected men with peripheral neuropathy	Acupuncture	Sham acupuncture	114	Valid in relieve of the pain and the mortality reduction, but the severe is not better than sham acupuncture	14 weeks
020	“Acupuncture Versus Paroxetine for the Treatment of Premature Ejaculation: A Randomized, Placebo-Controlled Clinical Trial”	Premature ejaculation	Acupuncture	Paroxetine	90	Valid	No
021	“Ear Acupuncture in the Treatment of Migraine Attacks: A Randomized Trial on the Efficacy of Appropriate Versus Inappropriate Acupoints”	Migraine attacks	Acupuncture on auricular point	Acupuncture on inappropriate auricular point	94	Valid	120 minutes
022	“Cost-Effectiveness of Acupuncture Care as an Adjunct to Exercise-Based Physical Therapy for Osteoarthritis of the Knee”	Osteoarthritis of the knee	Acupuncture care as an adjunct to exercise-based physical therapy	Education and excise	352	Valid	12 months
023	“A Randomised, Double-Blinded, Placebo-Controlled Study of Acupressure Wristbands for the Prevention of Nausea and Vomiting During Labour and Delivery”	Nausea and vomiting when delivery	Wrist band stimulative to acupoint	Wrist band nonstimulative to PC 6	340	Invalid	No mention
024	“Effect of Acupuncture on Allergen-Induced Basophil Activation in Patients with Atopic Eczema: A Pilot Trial”	Atopic eczema	Acupuncture	Blank	10	Valid	No mention
025	“Acupuncture to Treat Primary Dysmenorrhea in Women: A Randomized Controlled Trial”	Primary dysmenorrhea	Acupuncture	Sham acupuncture (2–4 cm beside acupoints and Streitberger sham acupuncture)	92	Valid	1 year
026	“Getting the Grip on Nonspecific Treatment Effects: Emesis in Patients Randomized to Acupuncture or Sham Compared to Patients Receiving Standard Care”	Emesis	Acupuncture	Sham acupuncture (2 cm beside acupoints and park sham acupuncture)	277	Both better than conventional therapy	No mention
027	“Perioperative Acupuncture and Postoperative Acupressure Can Prevent Postoperative Vomiting following Paediatric Tonsillectomy or Adenoidectomy: A Pragmatic Randomised Controlled Trial”	Postoperative vomiting	Acupuncture + conventional therapy	Conventional therapy	154	Valid	Yes
028	“The Effect of Acupuncture on Postmenopausal Symptoms and Reproductive Hormones: A Sham Controlled Clinical Trial”	Postmenopausal symptoms and reproductive hormones	Acupuncture	Sham acupuncture (the Streitberger sham acupuncture instrument)	55	Valid	No
029	“Efficacy of Acupuncture in Preventing Atrial Fibrillation Recurrences after Electrical Cardioversion”	Atrial fibrillation	Acupuncture	Sham acupuncture	54	Valid	12 months
030	“Acupuncture for the Induction of Labour: A Double-Blind Randomised Controlled Study”	Accouching	Acupuncture	Sham acupuncture (the Park sham acupuncture instrument)	125	Invalid	Yes
031	“Comparison between the Effects of Trigger Point Mesotherapy Versus Acupuncture Points Mesotherapy in the Treatment of Chronic Low Back Pain: A Short Term Randomized Controlled Trial”	Lumbago and backache	Lidocaine injection at acupoint	Lidocaine injection at trigger point	62	The trigger point mesoderm has good effect	12 weeks
032	“Relaxation Acupressure Reduces Persistent Cancer-Related Fatigue”	Cancer-related fatigue	Relaxation acupressure	High frequency of acupuncture and low frequency of acupuncture	43	Relaxation acupressure valid	No mention
033	“Delayed Effect of Acupuncture Treatment in OA of the Knee: A Blinded, Randomized, Controlled Trial”	OA of the knee	Acupuncture and conventional therapy	Sham acupuncture and conventional therapy	55	Valid	One month

**Table 3:** Jadad scores of the research.

Research	Randomization	Double blinding	Withdraws and drop outs	Jadad score
Park et al. [4]	Computer generated random table in a 1 : 1 ratio with block size 4, and using a sealed envelope	Patient blinded	2	5
Kashefi et al. [2]	Randomized	Single blinded	10	3
Penza et al. [5]	Randomized	Patient and examiner blinded	Not mentioned	2
Landgren et al. [6]	Not mentioned	Nurse and parents blinded	5	2
Smith et al. [7]	Computer generated randomization schedule	Statistician blinded	2	4
El-Deeb and Ahmady [8]	Not mentioned	Double blinded	Not mentioned	3
Scheewe et al. [9]	Not mentioned	Not mentioned	27%	2
Pastore et al. [10]	Block randomization	double blinded	14	4
Hamid et al. [11]	Not mentioned	Not mentioned	35	1
Paterson et al. [12]	Simple randomization	Statistician blinded	3,1	4
Rogha et al. [13]	Not mentioned	Not mentioned	9	1
Kim et al. [14]	Block randomization	open	3	3
Ecevit et al. [15]	Not mentioned	Not mentioned	Not mentioned	0
Do et al. [16]	Computer generated randomization	Not mentioned	1	2
Johnston et al. [17]	Block randomization	Open	1	3
Cox et al. [18]	Not mentioned	Not mentioned	1	0
Matsubara et al. [19]	Not mentioned	Not mentioned	Not mentioned	0
Gribel et al. [20]	Block randomization	Open	0	2
Shiflett and Schwartz [21]	Randomized	Patient and assessor blinded	19	4
Sunay et al. [22]	Simple randomization	Single blinded	0	3
Allais et al. [23]	Simple randomization	Single blinded	1	2
Whitehurst et al. [24]	Not mentioned	Researcher blinded	49	0
Sinha et al. [25]	Simple randomization	Double blinded	11	4
Pfab et al. [26]	Block randomization	Researcher blinded	0	3
Smith et al. [27]	Block randomization	Patient and assessor blinded	2	3
Enblom et al. [28]	Not mentioned	Assessor and nurse blinded	32	2
Liodden et al. [29]	Block randomization	Double blinded	32	4
Sunay et al. [30]	Not mentioned	Single blinded	2	2
Lomuscio et al. [31]	Not mentioned	Patient, assessor, and statistician blinded	0	3
Modlock et al. [32]	Block randomization	Patient, assessor, and statistician blinded	19	4
di Cesare et al. [33]	Block randomization	Assessor blinded	2	3
Zick et al. [34]	Computer generated randomization	Patient blinded	8	3
Lev-Ari et al. [35]	Not mentioned	Patient and assessor blinded	14	2

**Table 4:** Jadad score comparison of the acupuncture RCT methodologies in the 2011 SCI database.

Result	Total number of reports	Number of reports scoring at 1-2	Proportion (%)	Number of reports scoring at 3–5	Proportion (%)
Positive	23	9	39.13%	14	60.87%
Negative/placebo	10	4	40.00%	6	60.00%

**Table 5:** Intervention of 17 research reports with negative results or placebo effects.

Number	Disease	Intervention	Acupoint	Treatment frequency	Needling response	Qualification of acupuncturist
1	Constipation	Moxibustion	ST 23, ST 27	3 times/week	Unmentioned	Experience of more than five years
2	Chronic neuralgia	Sham-electroacupuncture after electroacupuncture for 12 weeks	ST36, SP6, LR3, BL60	1 time/week	Unmentioned	Unmentioned
3	Infantile colic	Acupuncture	LI 4	2 times/week	Unmentioned	Certificated nurse practitioner
4	Malposition	Moxibustion	BL 67	2 times/day	Unmentioned	Operation by patients themselves who are trained
5	Nausea and vomiting when delivery	Wrist band stimulating to acupoint	PC 6	1 time/week	Unmentioned	Unmentioned
6	Aids to delivery	Acupuncture	BL 67, LI 4, SP 6 and DU 20	2 times/day	Unmentioned	Acupuncturists and midwives, who often implement acupuncture treatment
7	Lumbago and backache	Lidocaine acupoint injection	GB30, BL31, BL52, GV3, ā shì points, GB34, GB 41, BL60, KI4, TE5	1 time/week	Unmentioned	3-year training and 8-year clinical experience
8	Knee osteoarthritis	Physical therapy plus verum acupuncture	Selecting 6 to 10 acupoints from SP9, SP10, ST 34, ST35, ST36, EX-LE5, GB 34, ā shì points, remote end: LI 4, SP 6, LR 3, ST 44, KI 3, BL 60, and GB 41	2 times/week	Yes	67 physical therapists reaching the lowest acupuncture level required by Acupuncture Association.
9	Knee joint pain of postmenopause women	Routine nursing plus acupressure	EX-LE4, ST35, SP 10, ST 34, ST 36, SP 9, GB 34, and EX-LE2	1 time/day	Unmentioned	Operation by patients themselves
10	Emergency treatment	Routine treatment plus acupuncture	Operation according to traditional Chinese medical standards	1 time/day	Unmentioned	Eligible acupuncturists having the experience of 6–22 years
11	Irritable bowel syndrome	Acupuncture	8-16 acupoints (no specific acupoints are mentioned)	1 time/week	Yes	Members approved by British Acupuncture Association
12	Woman health	Acupressure at SP 6	SP 6	Every day during menstrual period	Unmentioned	Trained about finger force practice
13	Polycystic ovarian syndrome	Electroacupuncture	Electroacupuncture: BL23 on two sides, BL 28, SP 6, and SP 9	Two times/week within the first four weeks, and one time/week within later four weeks	Unmentioned	Acupuncturists with the experience of five years
14	Emesis	Acupuncture	PC 6	Three times/week to two times/week	Yes	Unmentioned
15	Irritable bowel syndrome	Acupuncture	CV10, ST25, LR 3, SP4, PC6, ST 37	Unmentioned	Yes	2000-hour training and experience of more than four years
16	Low back pain	Acupuncture	Unmentioned	Two times/week	Yes	Physicians from various majors, trained about acupuncture more than 140 hours
17	Smoking cessation	Acupuncture	HT 7, PC 7, HT 8, KI 3, and KI 6	Three times/week	Yes	Unmentioned

**Table 6:** Intervention of 23 research reports with positive results.

Author	Disease	Intervention	Acupoint	Treatment frequency	Needling response	Qualification of acupuncturist
Smith et al. [7]	Infertility	Acupuncture	PC 6, PC 5, HT 5, HT 7	1 time/week	Yes	Certificated acupuncturist, experience of more than 14 years
El-Deeb and Ahmady [8]	Nausea and vomiting after cesarean	Electroacupuncture	PC 6	Single time	Unmentioned	Unmentioned
Scheewe et al. [9]	Bronchial asthma	Acupuncture	BL 13, CV 17, LU 7 and acupoint selection according to syndrome differentiation	3 times/week	Yes	Experience of many years
Hamid et al. [11]	Obesity	Auricular acupuncture	HT 7, CO4, CO1, HX1, CO17	2 times/day	Unmentioned	Unmentioned
Paterson et al. [12]	Psychoneurosis	Acupuncture and moxibustion	Not mentioned	According to syndrome differentiation	Yes	Member of British Medical Acupuncture Society
Rogha et al. [13]	Endogenous tinnitus	Acupuncture	TE 17, GB 2, SI 19, and TE 21	3 times/day	Unmentioned	Unmentioned
Kim et al. [14]	Low back pain	Cupping	BL 23, BL 24, BL 25	3 times/week	Unmentioned	3-year training and 6-year clinical experience
Ecevit et al. [15]	Analgesia	Acupuncture	EX-HN3	1 time/week	Unmentioned	Qualified acupuncturist
Kim et al. [14]	Cancer fatigue	Acupuncture	LI 4, SP 6, ST 36, KI 3	Not mentioned	Yes	Phd of TCM in the US, with 20 years of clinical experience
Cox et al. [18]	Motion sickness	Auricular acupuncture	TF4	Single time	Unmentioned	Unmentioned
Matsubara et al. [19]	Neck pain	Acupressure	GB 21, SI 14, SI 15, LI 4, LI 10, LI 11	Single time	Unmentioned	Unmentioned
Gribel et al. [20]	Cervical dilatation	Electroacupuncture	LI 4, ST 36, LR 3, SP 6, BL 23, BL 32	3 times/day	Unmentioned	With 20 years of clinical experience
Shiflett and Schwartz [21]	Peripheral neuropathy in ADIS patients	Acupuncture	SP 6, SP 7, SP 9, and acupoint selection according to syndrome differentiation	2 times/week	Unmentioned	Has received standardized training
Sunay et al. [22]	Premature ejaculation	Acupuncture	LI 4, ST 36, KI 3, LR 3, EX-HN3, CV 3	2 times/week	Unmentioned	Certificated and experienced acupuncturist
Allais et al. [23]	Migraine	Blunt needle	AT4	Single time	With	Experienced acupuncturist
Whitehurst et al. [24]	Knee osteoarthritis	Acupuncture	Conventional acupoints used for knee osteoarthritis	Not mentioned	Yes	Member of British Medical Acupuncture Society, physiotherapist
Pfab et al. [26]	Eczema	Acupuncture	LI 11, LI 4, ST 36, SP 10	2 times/week	Unmentioned	Experienced acupuncturist
Smith et al. [27]	Dysmenorrhea	Acupuncture	SP 4, CV 3, ST 29, SP 6, BL 32, SP 8	3 times/week	Yes	Certificated acupuncturist of CMASA
Liodden et al. [29]	Postoperative nause	Acupuncture + acupressure	PC 6	For 24 hours	Unmentioned	Experienced acupuncturist
Sunay et al. [30]	Perimenopausal syndrome	Acupuncture	ST 36, LI 4, KI 3, LR 3, EX-HN3, CV 3	2 times/week	Yes	Certificated acupuncturist of 6 years
Lomuscio et al. [31]	Atrial fibrillation	Acupuncture	PC 6, HT 7, BL 15	1 time/week	Unmentioned	Trained acupuncturist
Zick et al. [34]	Cancer fatigue	Acupressure	ST 36, SP 6, KI 3, LI 4, CV 6 GV 20, EX-HN3, HT 7, LR 3, SP 6	2 times a day, 3 times/week	Yes	Certificated acupuncturist with B.S. degree
Lev-Ari et al. [35]	Knee osteoarthritis	Acupuncture	GB 34, SP 5, ST 35, EX-LE5, LI 4, local points, ST 43, ST 34	2 times/week	Unmentioned	Unmentioned

**Table 7:** Control design in research report with negative or placebo result.

Number	Disease	Intervention of observation group	Intervention of control group
1	Constipation	Moxibustion	Heat insulation moxa-moxibustion (place a heat insulator under the true moxa-moxibustion)
2	Chronic neuralgia	Electroacupuncture for 12 weeks	Sham electroacupuncture (around the acupoint)
3	Infantile colic	Acupuncture	Without any measure
4	Malposition	Moxibustion	Without any measure
5	Nausea and vomiting when delivery	Wrist band stimulative to acupoint	Wrist band nonstimulative to PC 6
6	Accouching	Acupuncture	Sham acupuncture (the Park sham acupuncture instrument)
7	Lumbago and backache	Lidocaine injection at acupoint	Lidocaine injection at trigger point
8	Knee osteoarthritis	Physiotherapy and verum acupuncture	Physiotherapy and placebo acupuncture, or just physiotherapy
9	Knee joint pain of postmenopausal women	Usual care and acupressure	Usual care
10	Emergency treatment	Conventional treatment and acupuncture	Conventional therapy
11	Irritable bowel syndrome	Acupuncture	Sham acupuncture (unrelated to meridian points, without needling response)
12	Woman health	Acupressure at SP 6	Press the nonacupoint (not touch the tendo calcaneus in any meridian vessel on the back of leg)
13	Polycystic ovarian syndrome	Electroacupuncture	Stick on the skin by sham needle tubing, adjust the current to 0
14	Emesis	Acupuncture and moxibustion	Antinausea drug or sham acupuncture (two inches close to the acupoint by Park sham acupuncture instrument)
15	Irritable bowel syndrome	Acupuncture	Park sham needle, the nonacupoint close to acupoints
16	Low back pain	Acupuncture	Nonacupoint shallow penetration (1–3 mm)
17	Smoking cessation	Acupuncture	Nonacupoint shallow penetration (1–3 mm)

## References

[1] Liu H.-L., Zhang Y., Li J.-D., Cheng P., Wang L.-P. (2008). Design and practice of acupuncture placebo-controlled method in clinical studies of acupuncture. *Chinese Journal of Evidence-Based Medicine*.

[2] Kashefi F., Khajehei M., Ashraf A. R., Jafari P. (2011). The efficacy of acupressure at the sanyinjiao point in the improvement of women's general health. *The Journal of Alternative and Complementary Medicine*.

[3] Guo X. F., Wen Z. H., Lao Y. R. (2004). Quality assessment instruments of clinical trials and their application. *Chinese Journal of Evidence Based Medicine*.

[4] Park J. E., Sul J. U., Kang K., Shin B.-C., Hong K.-E., Choi S.-M. (2011). The effectiveness of moxibustion for the treatment of functional constipation: a randomized, sham-controlled, patient blinded, pilot clinical trial. *BMC Complementary and Alternative Medicine*.

[5] Penza P., Bricchi M., Scola A., Campanella A., Lauria G. (2011). Electroacupuncture is not effective in chronic painful neuropathies. *Pain Medicine*.

[6] Landgren K., Kvorning N., Hallström I. (2011). Acupuncture reduces crying in infants with infantile colic: a randomised, controlled, blind clinical study. *Acupuncture in Medicine*.

[7] Smith C. A., Ussher J. M., Perz J., Carmady B., De Lacey S. (2011). The effect of acupuncture on psychosocial outcomes for women experiencing infertility: a pilot randomized controlled trial. *Journal of Alternative and Complementary Medicine*.

[8] El-Deeb A. M., Ahmady M. S. (2011). Effect of acupuncture on nausea and/or vomiting during and after cesarean section in comparison with ondansetron. *Journal of Anesthesia*.

[9] Scheewe S., Vogt L., Minakawa S. (2011). Acupuncture in children and adolescents with bronchial asthma: a randomised controlled study. *Complementary Therapies in Medicine*.

[10] Pastore L. M., Williams C. D., Jenkins J., Patrie J. T. (2011). True and sham acupuncture produced similar frequency of ovulation and improved LH to FSH ratios in women with polycystic ovary syndrome. *The Journal of Clinical Endocrinology and Metabolism*.

[11] Hamid A., Parisa A., Zhao B. (2011). Effects of auricular acupuncture on anthropometric, lipid profile, inflammatory, and immunologic markers: a randomized controlled trial study. *Clinical Biochemistry*.

[12] Paterson C., Taylor R. S., Griffiths P. (2011). Acupuncture for ‘frequent attenders’ with medically unexplained symptoms: a randomised controlled trial (CACTUS study). *The British Journal of General Practice*.

[13] Rogha M., Rezvani M., Khodami A. R. (2011). The effects of acupuncture on the inner ear originated tinnitus. *Journal of Research in Medical Sciences*.

[14] Kim J.-I., Kim T.-H., Lee M. S. (2011). Evaluation of wet-cupping therapy for persistent non-specific low back pain: a randomised, waiting-list controlled, open-label, parallel-group pilot trial. *Trials*.

[15] Ecevit A., Ince D. A., Tarcan A., Cabioglu M. T., Kurt A. (2011). Acupuncture in preterm babies during minor painful procedures. *Journal of Traditional Chinese Medicine*.

[16] Do C. K., Smith C. A., Dahlen H., Bisits A., Schmied V. (2011). Moxibustion for cephalic version: a feasibility randomised controlled trial. *BMC Complementary and Alternative Medicine*.

[17] Johnston M. F., Hays R. D., Subramanian S. K. (2011). Patient education integrated with acupuncture for relief of cancer-related fatigue randomized controlled feasibility study. *BMC Complementary and Alternative Medicine*.

[18] Cox D. J., Singh H., Cox D. M. (2011). Effectiveness of acupressure and acustimulation in minimizing driving simulation adaptation syndrome. *Military Medicine*.

[19] Matsubara T., Arai Y.-C. P., Shiro Y. (2011). Comparative effects of acupressure at local and distal acupuncture points on pain conditions and autonomic function in females with chronic neck pain. *Evidence-Based Complementary and Alternative Medicine*.

[20] Gribel G. P. C., Coca-Velarde L. G., Moreira De Sá R. A. (2011). Electroacupuncture for cervical ripening prior to labor induction: a randomized clinical trial. *Archives of Gynecology and Obstetrics*.

[21] Shiflett S. C., Schwartz G. E. (2011). Effects of acupuncture in reducing attrition and mortality in HIV-infected men with peripheral neuropathy. *Explore: The Journal of Science and Healing*.

[22] Sunay D., Sunay M., Aydoğmuş Y. (2011). Acupuncture versus paroxetine for the treatment of premature ejaculation: a randomized, placebo-controlled clinical trial. *European Urology*.

[23] Allais G., Romoli M., Rolando S. (2011). Ear acupuncture in the treatment of migraine attacks: a randomized trial on the efficacy of appropriate versus inappropriate acupoints. *Neurological Sciences*.

[24] Whitehurst D. G. T., Bryan S., Hay E. M., Thomas E., Young J., Foster N. E. (2011). Cost-effectiveness of acupuncture care as an adjunct to exercise-based physical therapy for osteoarthritis of the knee. *Physical Therapy*.

[25] Sinha A., Paech M. J., Thew M. E., Rhodes M., Luscombe K., Nathan E. (2011). A randomised, double-blinded, placebo-controlled study of acupressure wristbands for the prevention of nausea and vomiting during labour and delivery. *International Journal of Obstetric Anesthesia*.

[26] Pfab F., Athanasiadis G. I., Huss-Marp J. (2011). Effect of acupuncture on allergen-induced basophil activation in patients with atopic eczema: a pilot trial. *Journal of Alternative and Complementary Medicine*.

[27] Smith C. A., Crowther C. A., Petrucco O., Beilby J., Dent H. (2011). Acupuncture to treat primary dysmenorrhea in women: a randomized controlled trial. *Evidence-Based Complementary and Alternative Medicine*.

[28] Enblom A., Lekander M., Hammar M. (2011). Getting the grip on nonspecific treatment effects: emesis in patients randomized to acupuncture or sham compared to patients receiving standard care. *PLoS ONE*.

[29] Liodden I., Howley M., Grimsgaard A. S. (2011). Perioperative acupuncture and postoperative acupressure can prevent postoperative vomiting following paediatric tonsillectomy or adenoidectomy: a pragmatic randomised controlled trial. *Acupuncture in Medicine*.

[30] Sunay D., Ozdiken M., Arslan H., Seven A., Aral Y. (2011). The effect of acupuncture on postmenopausal symptoms and reproductive hormones: a sham controlled clinical trial. *Acupuncture in Medicine*.

[31] Lomuscio A., Belletti S., Battezzati P. M., Lombardi F. (2011). Efficacy of acupuncture in preventing atrial fibrillation recurrences after electrical cardioversion. *Journal of Cardiovascular Electrophysiology*.

[32] Modlock J., Nielsen B. B., Uldbjerg N. (2010). Acupuncture for the induction of labour: a double-blind randomised controlled study. *BJOG*.

[33] di Cesare A., Giombini A., di Cesare M., Ripani M., Vulpiani M. C., Saraceni V. M. (2011). Comparison between the effects of trigger point mesotherapy versus acupuncture points mesotherapy in the treatment of chronic low back pain: a short term randomized controlled trial. *Complementary Therapies in Medicine*.

[34] Zick S. M., Alrawi S., Merel G. (2011). Relaxation acupressure reduces persistent cancer-related fatigue. *Evidence-Based Complementary and Alternative Medicine*.

[35] Lev-Ari S., Miller E., Maimon Y. (2011). Delayed effect of acupuncture treatment in OA of the knee: a blinded, randomized, controlled trial. *Evidence-based Complementary and Alternative Medicine*.

[36] Foster N. E., Thomas E., Barlas P. (2007). Acupuncture as an adjunct to exercise based physiotherapy for osteoarthritis of the knee: randomised controlled trial. *British Medical Journal*.

[37] Zhang Y., Shen C.-L., Peck K. (2012). Training self-administered acupressure exercise among postmenopausal women with osteoarthritic knee pain: a feasibility study and lessons learned. *Evidence-Based Complementary and Alternative Medicine*.

[38] Painovich J., Herman P. M. (2012). Acupuncture in the inpatient acute care setting: a pragmatic, randomized control trial. *Evidence-Based Complementary and Alternative Medicine*.

[39] Forbes A., Jackson S., Walter C., Quraishi S., Jacyna M., Pitcher M. (2005). Acupuncture for irritable bowel syndrome: a blinded placebo-controlled trial. *World Journal of Gastroenterology*.

[40] Lembo A. J., Conboy L., Kelley J. M. (2009). A treatment trial of acupuncture in IBS patients. *The American Journal of Gastroenterology*.

[41] Haake M., Müller H.-H., Schade-Brittinger C. (2007). German Acupuncture Trials (GERAC) for chronic low back pain: randomized, multicenter, blinded, parallel-group trial with 3 groups. *Archives of Internal Medicine*.

[42] Hyun M.-K., Lee M. S., Kang K., Choi S.-M. (2010). Body acupuncture for nicotine withdrawal symptoms: a randomized placebo-controlled trial. *Evidence-Based Complementary and Alternative Medicine*.

[43] He J. S. (2002). Discussions on acupuncture clinical education in new century. *Shanghai University of Chinese Medicine*.

[44] Cai N. Z., Ma Z. Y. (1986). Effect to renal uresis function by shenshu point acupuncture. *Shanghai Journal of Acupuncture-Moxibustion*.

[45] Park J., White A., Stevinson C., Ernst E., James M. (2002). Validating a new non-penetrating sham acupuncture device: two randomised controlled trials. *Acupuncture in Medicine*.

[46] Wang Y., Liu Z.-S. (2011). Thinking on control methods in acupuncture trials. *Chinese Journal of Evidence-Based Medicine*.

[47] Ceccherelli F., Rigoni M. T., Gagliardi G., Ruzzante L. (2002). Comparison of superficial and deep acupuncture in the treatment of lumbar myofascial pain: a double-blind randomized controlled study. *The Clinical Journal of Pain*.

[48] Liu Y. (2001). *Collection of Chinese Extra-Point*.

[49] Wang Y. Z. (2012). Critique and reflection on methodology of clinical research. *Chinese Acupuncture and Moxibustion*.

[50] Liu Z.-S., Cai Y.-Y. (2010). Thinking on methodologies and problems existed in clinical study of acupuncture and moxibustion. *Chinese Acupuncture & Moxibustion*.

[51] Huang M. F., Yu D. C. (2004). Dependability test on placebo control acupuncture method. *Chinese Acupuncture-Moxibustion*.

[52] Liu J., Wang J. Y., Liu J. L. (2007). Placebo control in the design of acupuncture clinical trials. *Acupuncture Research*.

[53] Liu W. H. (2013). Emphasize curative effect advantage—our target of acupuncture clinical research. *Chinese Acupuncture-Moxibustion*.

